# Integrating gene expression, genomic, and phosphoproteomic data to infer transcription factor activity in lung cancer

**DOI:** 10.1093/nargab/lqaf068

**Published:** 2025-05-30

**Authors:** Chiara Carrino, Gerardo Pepe, Luca Parca, Manuela Helmer-Citterich, Pier Federico Gherardini

**Affiliations:** Department of Biology, University of Rome “Tor Vergata”, 00133 Rome, Italy; PhD Program in Cellular and Molecular Biology, Department of Biology, University of Rome “Tor Vergata”, 00133 Rome, Italy; Department of Biology, University of Rome “Tor Vergata”, 00133 Rome, Italy; Italian Space Agency, Via del Politecnico snc, 00133 Rome, Italy; Department of Biology, University of Rome “Tor Vergata”, 00133 Rome, Italy; Department of Biology, University of Rome “Tor Vergata”, 00133 Rome, Italy

## Abstract

Transcription factors (TFs) are key regulators of cellular gene expression programs in health and disease. Here we set out to integrate genomic, transcriptomic, and phosphoproteomic data to characterize TF activity in lung adenocarcinoma patients. Using expression data from patient samples and genomic information on TF binding to super-enhancers, starting from a list of 1667 human TFs we calculated a patient-specific activity score and identified 34 with perturbed activity in the cancer samples, as evidenced by the expression of their direct targets. We then leveraged phosphoproteomic data on the same samples to identify phosphorylation events that modulate TF activity. This novel data integration approach to TF characterization led to the identification of ERG as a key regulator in lung adenocarcinoma whose activity strongly correlates with patient survival.

## Introduction

Transcription factors (TF) play an important role in tumorigenesis, serving as central regulators for the activity of the large and complex expression programs that ultimately govern cell fate [1]. Various studies have investigated their role in different diseases, with the goal of deriving prognostic and diagnostic biomarkers. For instance, TFs of the E2F family regulate tumor growth and metastasis in oral cancers [2] and represent diagnostic and prognostic biomarkers in that disease. Moreover, a recent study of TCGA lung cancer data revealed that higher expression levels of BACH1 are associated with poor survival [3]. Similarly, a set of six TFs was used to develop a signature that predicts patient survival in ovarian cancer [3, 4]. TFs are also being explored as therapeutic targets, as in the case of STAT family members [5]. The role of STAT TFs in tumorigenesis is complex, with different STAT family members exhibiting varying effects on cancer progression. Research in cell lines suggests that inhibition of STAT3 activity in cancer cells could prevent or even reverse resistance to anti-cancer drugs [6] Meanwhile STAT2, a TF and downstream mediator of type I interferon (IFN-I) signaling, can either inhibit or enhance tumorigenesis, with its effects being dependent on the distinct microenvironment in each cancer type [7]. Given the critical role that TFs play in regulating cellular behaviour, the inference of their activity from transcriptomic data is an active area of research [8]. A common approach is based on the identification of genes whose expression is correlated with that of a TF of interest, therefore representing its putative targets [9, 10]. The interactions between TFs and their targets are followed by a confidence score, based on the number of supporting evidence, as in Dorothea [2, 11] and can offer to researchers pre-computed regulons (set of genes regulated by the same TF) by integrating multiple sources, such as ChIP-Seq experiment results and TF-binding prediction on gene promoters.Other resources such as CollecTRI [12] derive regulons from the integration of literature data and computational methods that infer the sign (i.e. activatory versus inhibitory) of the interaction between target genes and TFs from expression data. Methods such as ARACNE-ap [13] and *corto* [10] leverage the coexpression of TFs and target genes to identify regulons in a specific dataset provided by the user. These methods calculate the correlation between the expression of TFs and other genes in the dataset, and then apply a threshold to select significant relationships. These methods are therefore purely data-driven and do not rely on databases of interactions mined from the literature. While powerful, this strategy has several limitations. First, it does not take into account the fact that TF binding motifs must be present in the enhancers and super-enhancers that regulate the expression of a gene, for the TF to exert its effects. In particular super enhancers are large clusters of enhancers with high levels of transcriptional activity that regulate genes involved in key cellular processes, such as cell identity and disease development. They are typically marked by high concentrations of TFs and cofactors, and their disruption can have significant effects on gene expression [14, 15]. Indeed, physical binding to the genome distinguishes direct targets of a TF from genes whose expression is correlated to that of the TF due to second-order effects, i.e. genes that are regulated by a direct target of the TF. Second, the activity of a TF is not exclusively controlled by its expression level. Indeed, several additional regulatory layers modulate the function of a TF, including its interaction with other proteins and its post-translational modifications. With respect to the latter, phosphorylation is a known key driver of TF activity which can be investigated thanks to recent advancements in phosphoproteomic techniques. Multiple studies have investigated the role of TF phosphorylation in the context of cancer [10, 16]. For instance the phosphorylation of STAT3 causes a downstream change in the expression of genes that leads to uncontrolled proliferation in cervical cancer [17]. Similarly, the phosphorylation of the TFs TFEB and eTFE3 regulates autophagy and lysosomal activity in lung cancer [18]. To address the above-mentioned issues, here we introduce an analytical pipeline aimed at comprehensively characterizing TF activity through the integration of genomic, transcriptomic, and phosphoproteomic data. This approach was deployed in the context of lung adenocarcinoma by leveraging a multi-omic dataset derived from tumor and matched normal adjacent tissue (NAT) samples [19]. Beyond genome sequencing, transcriptomics and phosphoproteomics provide a unique opportunity to investigate the behavior of cancer cells, and to identify predictive and prognostic biomarkers as well as new therapeutic targets [20]. Accordingly, we show that the integration of multiple layers of information leads to a more informative characterization of TF activity, which ultimately resulted in the identification of strong correlates of patient survival.

## Materials and methods

### Dataset details

The normal adjacent and tumor samples phosphoproteomics data used in this paper were collected as pre-processed data from the Proteomic Data Commons (PDC) repository (https://pdc.cancer.gov), from a study conducted as part of the Clinical Proteomic Tumor Analysis Consortium (CPTAC) project, published in 2020 [19] (PDC000149). Transcriptomics data (RNA-seq pre-processed counts) for this study were collected from the Genomic Data Commons portal (GDC) (https://www.ncbi.nlm.nih.gov/projects/gap/cgi-bin/study.cgi?study_id= phs001287.v5.p4). Only matched patient samples present in transcriptomic and phosphoproteomic datasets were considered, resulting in 200 paired samples analyzed in this study.

### Inference of transcription factors activity from genomic and transcriptomic data

In order to infer the activity of TFs and their targets from gene expression data, we used *corto* (version 1.2.2), a method to infer gene regulatory networks and perform master regulator analysis (MRA, described in details later in the text) available as an R package (https://github.com/federicogiorgi/corto) [10]. A list of 1667 human TFs, derived from Dorothea [11], The Human Transcription Factors database [21], and ChEA [22] was used as input for the analysis. Dorothea is a curated collection of human and mouse regulons, while ChEA is a database of TFs and associated target genes derived from the integration of different ChIP-X experiments. To infer the list of targets for each TF we used RNA-seq data from the 100 healthy tissue samples normalized using the variance stabilizing transform (VST), as described in the *corto* documentation, with default parameters (nthreads = 8, *P*-value threshold = 1e-8, nbootstraps = 1000). Also as suggested in the documentation, TFs with <20 direct targets were excluded from further analysis. We then filtered the target lists of each TF to only include direct targets, by leveraging the information contained in SEanalysis [23], a database of super enhancers which aggregates a large amount of data including experimental ChIP-seq data, detection of DNA-binding sequence motifs, and pathway information. To this end, SEanalysis was first filtered to only include data from lung samples. We then searched for enhancers bound by the 1010 TFs whose targets inferred by *corto* are expressed in our data, retaining 1156 of the original 331 551 enhancers contained in the database. We filtered the regulons obtained by *corto* to only include target genes regulated by enhancers that are directly bound by the corresponding TF, as reported in SEanalysis. The final list of 34 TFs, SEs, and direct target genes is available in the Supplementary Table S1. Finally, to infer the activity of each TF in tumor samples given its list of targets, we performed an MRA using the matched tumor samples with the *mra* function in the *corto* package. The input to the *mra* function consists of the two expression matrices for the tumor and NAT samples, as well as the regulon that describes the targets of the TF under analysis. Here, the regulon was derived from the expression data of the NAT samples alone. The MRA analysis produces a Normalized Enrichment Score (NES) for each TF, which reflects its level of activity in the tumor samples, and depends on the expression level of its targets, resulting in an activity score derived from the aggregated data of all the samples. The NES is positive if the positive targets of a TF tend to be upregulated, and the negative targets tend to be downregulated, and negative in the opposite case (positive targets downregulated and negative targets upregulated). The output of the *corto* MRA analysis before and after the selection of direct target genes, together with the sample-specific activity scores is available in Supplementary Table S1. To derive a sample-specific TF activity score we calculated, for each sample, the sum of the variance-stabilized expression of its direct positive targets, minus the sum of the expression of its negative targets. The code was developed using R 4.4.1 and Python 3.11.3.

### Gene Set Enrichment Analysis

Gene Set Enrichment Analysis (GSEA) on TFs clusters was performed with GSEApy (v 1.1.4) [24] in Python, specifically by employing the “enrichr” module [25].

### Identification of phosphorylations that modulate transcription factor activity

To identify phosphorylation events that are predictive of TFs activity we built a Least Absolute Shrinkage and Selection Operator (LASSO) regression model using the implementation available in the *glmnet* (v. 4.1.8) R package and *caret* (v. 7.0.1). We divided the phosphorylation dataset in a training set and a test set with 80% and 20% observations, respectively, by using the function createDataPartition. Here, LASSO was used as a feature selection method, to identify, among the many phosphorylation events present in the dataset, the ones that are most closely associated with TF activity. The model uses the log2 fold change in phosphorylation between the paired tumor and NAT samples as predictors, and the patient-specific TF activity scores as the outcome variable. We constructed a different model for each one of the 34 TF in our final dataset (see above). For each TF, only phosphorylation sites detected in all patient samples and located on proteins participating in the same biological pathways as the TF were used as predictors. Pathway membership was determined by using the Canonical Pathway collection of MSigDB [26], which integrates information from multiple databases including BioCarta [27], KEGG [28], PID [29], Reactome [30], and WikiPathways [31]. A five-fold cross-validation was used to identify the optimal lambda value. We employed the R function trainControl() to set the parameters for the cross-validation performed in the train() function. The LASSO results are reported in Supplementary Table S2. For three TFs, it was not possible to create a model. More specifically, for FOXJ3 and SP2, no phosphorylations were identified in the phosphoproteomic data that involved substrates participating in the same biological pathways as the TFs. For FOXO3, the range of lambda regularization values we tested did not produce any model with non-zero coefficients. To derive a sample-specific measure of phosphorylation pathways activity in tumor samples, phosphosites that were identified by LASSO as being predictive of TFs activity were divided according to the sign (positive versus negative) of the model coefficients. The log2 fold change values of phosphorylations sites with positive coefficients were then summed to derive a phosphorylation-based TF activity score; the same was done with negative coefficients.

### Inference of kinase activity from phosphoproteomic data

We employed RoKAI [32], a web tool for the inference of kinase activity from paired phosphoproteomic profiles of NAT samples and tumor samples. We selected from the initial dataset, only matched samples for which both phosphoproteomic and transcriptomic data were available, resulting in 200 samples, 100 NAT and 100 paired tumor samples. Then, for every patient, we calculated the log2 fold change between tumor and NAT samples. We obtained a patient specific kinases activity measure, by giving in input to RoKAI the phosphorylation fold change values. We further investigated the role of three kinases: MTOR, CSNK2A1, and PRKCE which are involved in the ETS Transcription Factor ERG (ERG) signaling pathway (for further information, see “Results” section). The calculated kinase activity values were retained if their false discovery rate (FDR)-adjusted *P*-value, as calculated by RoKAI, was statistically significant (FDR ≤ 0.05). Kinase activity scores and associated FDR values are available in Supplementary Table S3. For MTOR, PRKCE, and CSNK2A1, we identified 83, 82, and 95 significant patient-specific activity measures, respectively.

## Results

### Overall view of the workflow

**Figure 1.**
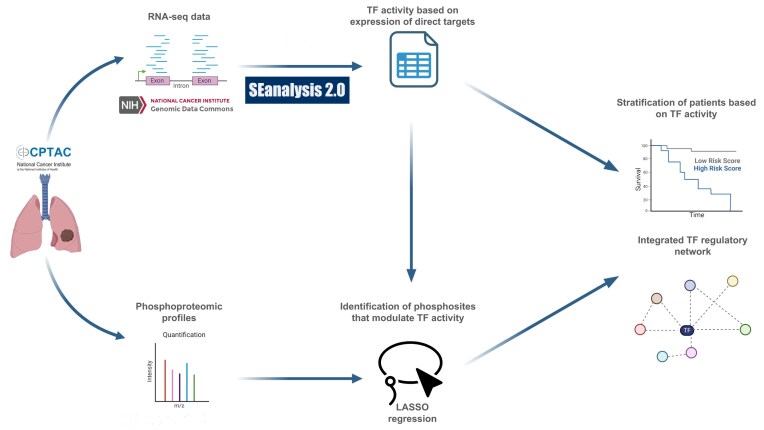
This figure outlines the key steps of our analysis. Starting with transcriptomic data from CPTAC, we leveraged *corto* and SEanalysis 2.0 to calculate a sample-specific TF activity measure and evaluate its association with patient survival. In the following steps, a LASSO regression model was used to identify, from phosphoproteomic data collected on the same samples, phosphorylation events that modulate TF activity and are themselves independently associated with patient survival. Finally, integration of these two layers of information led to the identification of ERG and its associated regulatory network as a key determinant of patient outcomes.

Figure 1 provides an overall view of the workflow. We sought to comprehensively characterize TF activity in lung cancer to identify TFs whose activity was altered in cancer samples, and potentially associated with patient survival. We first applied the *corto* method [10] on transcriptomic data from a cohort of 100 lung adenocarcinoma patients [19]. This cohort was selected because both transcriptomic and phosphoproteomic data were available for the same matched tumor and NAT samples. Using genomic information on TF binding available in the SEanalysis database [33], we then identified transcriptional networks (henceforth “regulons”) with perturbed activity. We then integrated phosphoproteomic data on the same samples to identify phosphorylation events that modulate the activity of these TFs by using a LASSO model for feature selection. The integration of multiple layers of information led to the identification of the ERG TF as a key regulator, whose activity is strongly associated with patient survival.

### Transcription factors activity scores in lung cancer

We first sought to identify networks (henceforth “regulons”) of TFs target genes whose activity was altered in lung adenocarcinoma. To this end, we inferred regulons by applying the *corto* method [10] on expression data from NAT samples in a cohort of lung adenocarcinoma patients [19].

We then performed an MRA which produces a NES for each TF, describing whether its target genes are significantly up- or down-regulated in tumor samples. The NES can be interpreted as a proxy for the overall change in TF activity over the entire set of tumor samples, when compared with NAT.

Starting from a literature-derived list of 1667 known human TFs (see “Materials and methods” section), we identified 1193 TFs with am associated NES, of which only 1010 transcriptional networks that are significantly (NES with a *P*-value ≤ 0.05) perturbed in tumor samples.

**Figure 2.**
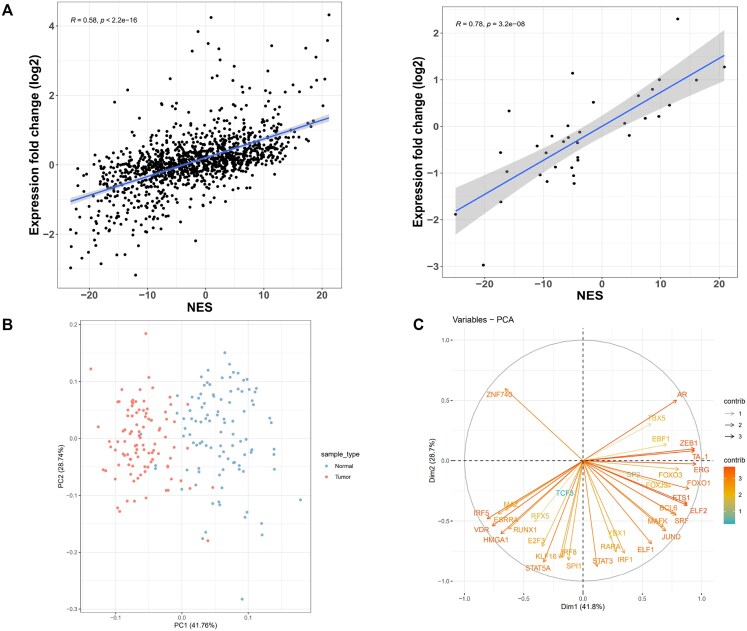
(**A**) Left: Scatterplot of TFs NES (*x*-axis) versus the gene expression log2 fold change of the TF between tumor and paired NAT samples obtained by DESeq2 (*y-*axis). Every point in the scatterplot is a TF. Right: the same plot for the 35 TFs with direct target genes that are perturbed in cancer samples. (**B**) PCA plot of sample specific TF activity scores for tumor and NAT samples. Samples from the two conditions are shown in different colors. The first two principal components explain ∼70% of the variance in the dataset (**C**) Graphical representation of the contributions of variables to principal component 1 (PC1) and principal component 2 (PC2). The color scale and the line size indicate the contributions (“contrib” in the legend) of each variable to the principal components.

As expected, the NES score of the 1193 TFs, and hence the TF activity, is positively correlated (Pearson correlation coefficient = 0.58 and a *P*-value < 2.2 × 10^−16^) with the differential expression of the TFs in the tumor samples (Fig. 2A). The correlation was computed with the stat_cor() function in R programming language. The associated *P*-value was computed based on a *t*-distribution with *n* − 2 degrees of freedom. In order to distinguish direct targets of a TF from other genes whose expression is correlated to that of the TF due to indirect upstream or downstream effects, we filtered the TF regulons to only include genes for which we could obtain evidence of direct TF binding in lung tissue from the SEanalysis database. Starting from the SEanalysis database, we searched for SEs bound by the 1010 TFs whose activity is significantly perturbed, according to the above analysis. We found 254 TFs binding 1.156 different SEs which are modulating the expression of 1.282 target genes (henceforth called “direct targets”). This step allowed us to filter regulons to only include genes that are directly targeted by the TF. Thirty-five of these TFs yielded an NES value in the MRA analysis, with 34 having an NES associated with a significant *P*-value (≤ 0.05). A summary of the TFs which were filtered at each step of the analysis is included in Supplementary Table S4.

For this set of 35 TFs and their direct targets, we observed a stronger correlation between TFs activity (as defined by the NES score from the MRA analysis) and the differential expression of the TFs themselves (Pearson correlation coefficient.78 and a *P*-value = 3.2 × 10^−08^ see Fig 2A).

### Sample-specific TF activity scores

The above analysis identifies TFs whose activity is significantly perturbed in tumor samples, as evidenced by the expression of their direct targets. However, we expect the pattern of TFs activation to vary in different subsets of patients. Accordingly, in order to investigate the heterogeneity of TF activity, we derived a sample-specific TF activity score by summing the expression value of their positive direct targets and subtracting the sum of the expression of the negative direct targets in individual samples, following VST. We used principal component analysis (PCA) to identify linear combinations of TF activity scores that capture most of the variation between samples (see Fig. 2B). Tumor and normal samples are clearly separated along PC1. ERG, TAL1, ZEB1, FOXO1, ELF2, ETS1, IRF5, FOXO3, AR, and SRF are among the TFs whose activity most strongly separates tumor from normal samples as evidenced by their loadings on PC1 (Fig. 2C and Supplementary Fig. S1).

### Patterns of transcription factor activities

**Figure 3.**
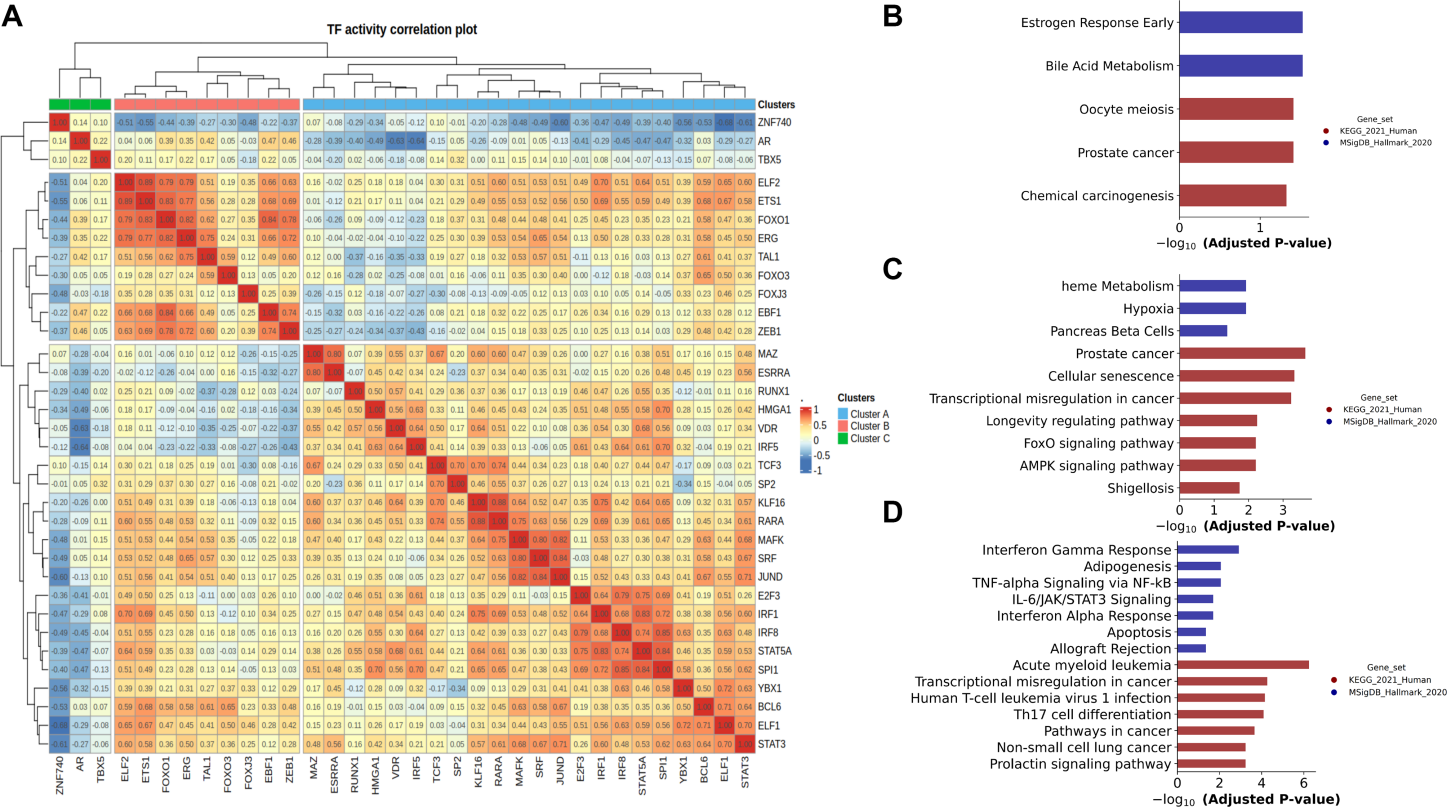
(**A**) Heatmap depicting the correlation of TF activity scores in tumor samples. (**B**) GSEA of the green cluster (cluster C in the figure legend) in the correlation plot. (**C**) Enrichment analysis of the red cluster (cluster B in the figure legend) in the correlation plot. (**D**) Enrichment analysis of the blue cluster (cluster A in the figure legend) in the correlation plot.

To identify possible functional relationships among TF, we calculated a matrix of correlation values between their sample-specific activity scores (Fig. 3A). After visual inspection of the dendrogram, we identified three clusters of TFs, which were further analyzed by GSEA with GSEApy [24] using gene sets derived from KEGG [28] and MSigDB [26]. The first cluster is composed of ZNF740, AR, and TBX5, which are implicated in prostate cancer (Fig. 3B). A second cluster (ELF2, ETS1, FOXO1, ERG, TAL1, FOXO3, FOXJ3, EBF1, and ZEB1) includes TFs regulating a diverse set of biological processes, including the transcriptional misregulation in cancer KEGG pathway and the KEGG prostate cancer pathway (Fig. 3C). The third cluster contains TFs (MAZ, ESRRA, RUNX1, HMGA1, VDR, IRF5, TCF3, SP2, KLF16, RARA, MAFK, SRF, JUND, E2F3, IRF1, IRF8, STAT5A, SPI1, YBX1, BCL6, ELF1, and STAT3) that are involved in regulating the immune response, particularly the interferon γ pathway. TFs of this cluster are also enriched in the KEGG database for participating in a collection of genes annotated as “nonsmall cell lung cancer” (Fig. 3D).

### Association between transcription factor activity and patient survival

**Figure 4.**
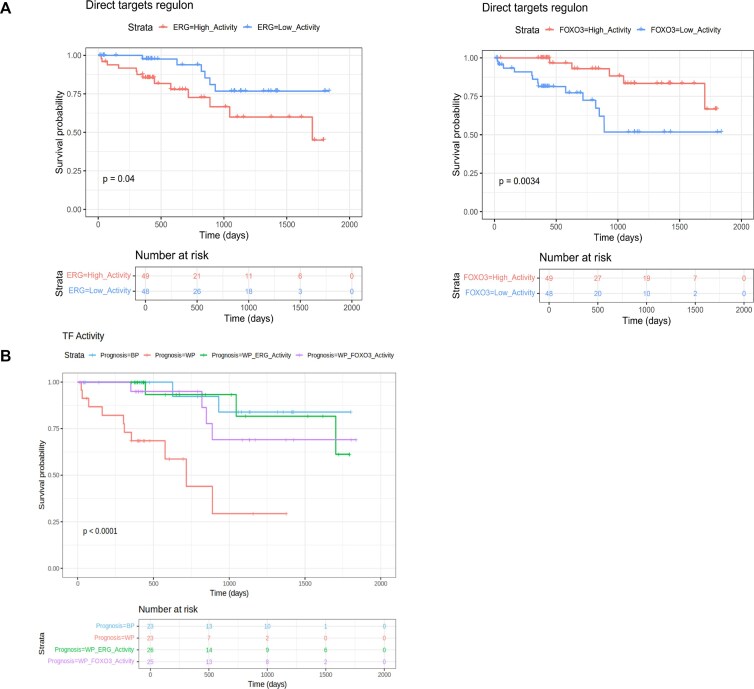
(**A**) Kaplan–Meier survival curves for the two TFs whose activity in tumor samples is significantly associated with patient survival. Patient strata (high versus low) were defined according to the median of the TF activity scores. The two curves refer to ERG and FOXO3 activity scores calculated from the direct targets only. (**B**) Kaplan–Meier survival curves for patients stratified according to whether they are in the worst prognosis group for none (Prognosis = BP), one (Prognosis = WP_ERG_Activity, Prognosis = WP_FOXO3_Activity), or both (Prognosis = WP) of the TFs.

We next performed a Kaplan–Meier analysis to investigate whether the level of activity of any of the 34 TFs in the final dataset was associated with patient survival. For each TF, we defined two strata of patients (high versus low) according to the median of the activity values. The activity of two TFs, FOXO3 and ERG, was found to be significantly associated with survival (*P*-value ≤ 0.05) (Fig. 4A). Interestingly, both TFs show a significant association with survival (*P*-value ≤ 0.05) when the TFs direct targets are used to infer TF activity, while no significant association using all the targets inferred by *corto* was found (Supplementary Fig. S2). This suggests that the integration of this additional layer of evidence, better captures the underlying activity of the TF. ERG (ETS TF ERG) is a TF involved in the regulation of multiple processes, including cell proliferation, differentiation, and angiogenesis. This TF is well known for its role in oncogenic gene fusion products in multiple cancers including prostate cancer (TMPSSR2-ERG and NDRG1-ERG), in Ewing’s sarcoma (EWS-ERG) and acute myeloid leukemia (FUS-ERG) [34]. In patients affected by prostate cancer, overexpression of ERG is associated with disease progression [35]. We also calculated the activity score of ERG in a separate cohort of lung cancer patients from TCGA and similarly used it for patient stratification, obtaining a *P*-value of 0.067 in the Kaplan–Meier analysis (Supplementary Fig. S6). FOXO3 (Forkhead Box O3) regulates the expression of apoptosis related genes. FOXO3 has a role in different diseases such as Rhabdomyosarcoma and secondary acute leukemia [36], and it is also a regulator of regulatory T-cell differentiation [37]. We then set out to investigate whether integrating the information from the two TFs leads to a stronger prediction of patient survival. To this end we stratified patients according to the number of TFs for which they are in the worst prognosis group (see Supplementary Fig. S3). Patients in the bad prognosis group for both FOXO3 and ERG exhibit worse outcomes (*P*-value 2.68 × 10^−5^), compared with ones that are classified as having a bad prognosis based on a single TF (ERG *P*-value = 0.04 and FOXO3 *P*-value = 0.0034) (Fig. 4). This analysis demonstrates that this set of two TFs captures multiple underlying aspects of the disease biology, whose integration leads to a stronger prognostic model.

### Modulation of transcription factor activity by phosphorylation

**Figure 5.**
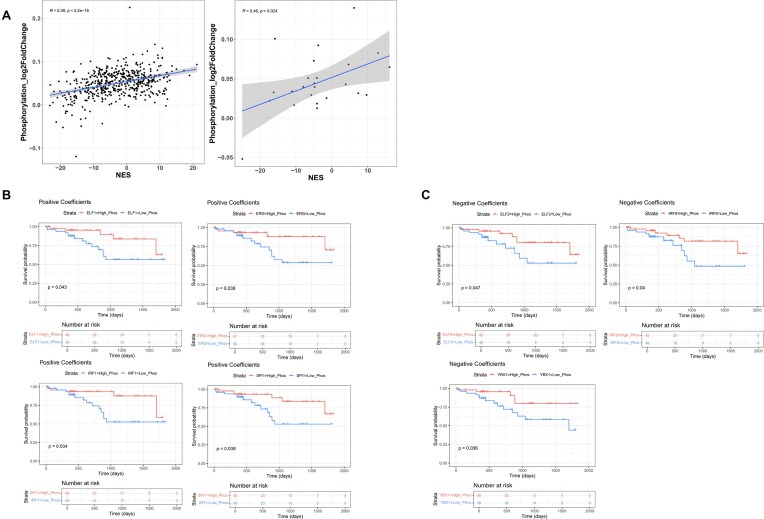
(**A**) Left: Scatterplot of NES scores derived by *corto* (*x-*axis) versus phosphorylation log2 fold changes between tumor and NAT samples (*y-*axis). Right: the same plot for 34 TFs that are significantly perturbed in cancer samples, as revealed by the expression of their direct targets. Here, the NES was calculated using the direct targets only. Kaplan–Meier analysis for TF phosphorylation scores based on positive (**B**) and negative (**C**) regression coefficients. Patient strata were defined using the median of the phosphorylation scores. Only significant results (*P* ≤ 0.05) are displayed.

Analysis of the phosphoproteomic data showed the overall activity of TFs in tumor samples, as represented by the NES in the MRA analysis, to be correlated with their phosphorylation levels (Fig. 5A). To further investigate how the phosphorylation of other proteins upstream and downstream of a TF (i.e. beyond the TF itself) modulates its activity, we used sample-level data to build a LASSO regression model, relating TF activity (response variable) with the phosphorylation of all the proteins that participate in the same biological pathways (predictor variables, see “Materials and methods” section).

Here, LASSO regression was not used with the goal of developing a generalized predictor of TF activity, but instead as a feature selection mechanism to identify, among all the many possible phosphosites, the ones whose abundance is more strongly linked to TF activity. From the initial list of 34 TFs we selected, for every TFs, the set of phosphorylation sites located in proteins that share at least one pathway from the MSigDB canonical pathway collection with the TF. We thus obtained 31 LASSO models (3 TFs did not yield any valid models), involving a total of 336 unique predictive phosphorylations. The sets of phosphorylations that are predictive of each TF activity are available in the Supplementary Table S2. Similarly to what we did for TF activity scores, we investigated whether a combined score derived from the phosphorylation of these proteins is associated with patient survival. For each TF, the LASSO model coefficients were divided in two groups, according to their sign (positive versus negative). We then summed the log2 fold change values between paired tumor and NAT samples for the phosphorylation sites associated with positive or negative coefficients. The resulting phosphorylation score defines a measure of TFs activity, based on the phosphorylation of its upstream regulators and downstream targets. A Kaplan–Meier analysis identified four TFs in the positive coefficients group (ELF1, ERG, IRF1, and SPI1) (see Fig. 5B) and three in the negative group (ELF2, IRF5, and YBX1) (Fig. 5C). This analysis reveals that phosphorylations which are involved in the regulation of TF activity, also correlate with patient prognosis. These findings highlight the potential significance of the pathways in which these phosphorylations and the corresponding TFs are implicated.

This analysis demonstrated that the phosphorylation sites identified by LASSO as predictive of ERG activity, are associated with patient survival, similar to the activity of ERG itself (see Table 1). This suggests a key role for ERG and for the activity of its upstream regulators and downstream targets in the biology of the disease. All of these phosphorylations are annotated in the PhosphoSiteplus database [38] or in the SIGNOR database [39] for their role in different diseases, including nonsmall cell lung cancer (NSCLC). Specifically, the phosphorylation of T710 on PRKCE is known to be essential for its activation. This modification is putatively mediated by the MTOR kinase [40] and represents a positive regulator of cell growth. In fact, the PI3K/AKT/mTOR signaling cascade is crucial in driving disease progression and tumor development, and it is often activated in NSCLC. This pathway promotes cell survival, continuous migration, and angiogenesis, as well as conferring growth signal independence, enhanced invasiveness, and resistance to both metastasis and anti-growth signals [41]. Additionally, both S609 on FGA and S1943 on MYH9 are phosphorylated by CSNK2A1 [42, 43], a subunit of the protein kinase CK2 which phosphorylates many TFs in tumor cells [44]. CSNK2A1 was found to be significantly over-expressed in gastric cancer cells compared to normal gastric epithelium [45]. Stable overexpression of CSNK2A1 in SNU216 cells resulted in a marked increase in cellular proliferation, invasion, and migration, suggesting its involvement in driving oncogenic processes. Furthermore, it was demonstrated that CSNK2A1 regulates epithelial-to-mesenchymal transition (EMT) and enhances the invasiveness of gastric cancer cells through the PI3K–Akt–mTOR signaling pathway [45]. These findings underscore the critical role of CSNK2A1 and mTOR in modulating the ERG signaling pathway, highlighting their potential as key targets in LUAD as well. The remaining phosphorylations are implicated in cytoskeleton assembly and cell signaling transduction (IQGAP1) [46], and cellular migration (NEXN) [47]. All of the aforementioned phosphorylations play critical roles in regulating key cellular functions, particularly cell motility, adhesion, and migration. These processes are essential for endothelial cell behavior, vasculature formation, and remodeling. Phosphorylation events, such as those in MYH9 (S1943), MYH11 (S1954), ALB (S82, T551), NEXN (S16), PRKCE (T710), and others, contribute to the regulation of actin dynamics, cell–cell junctions, and survival pathways within endothelial cells [46, 48, 49]. When coupled with alterations in ERG activity, these disruptions in endothelial cell signaling could lead to a breakdown in the delicate balance required for normal angiogenesis, ultimately contributing to aberrant vasculature growth.

**Table 1.** Phosphorylations identified by LASSO as predictive of ERG activity in tumor samples

Substrate	Phosphorylation site	LASSO coefficient	Kinase
MYH9	S1943	1.209	CSNK2A1
MYH11	S1954	1.100	–
ALB	S82	0.881	–
NEXN	S16	0.637	–
PRKCE	T710	0.503	MTOR
MYH11	S23	0.282	–
FGA	S609	0.203	CSNK2A1
ALB	T551	0.122	–
SSR3	S105	0.100	–
PRKCD	S683	0.091	–
FHOD1	S549	0.044	–
CTNND1	S847	−1.208	–
IQGAP1	S330	−1.380	–

**Figure 6.**
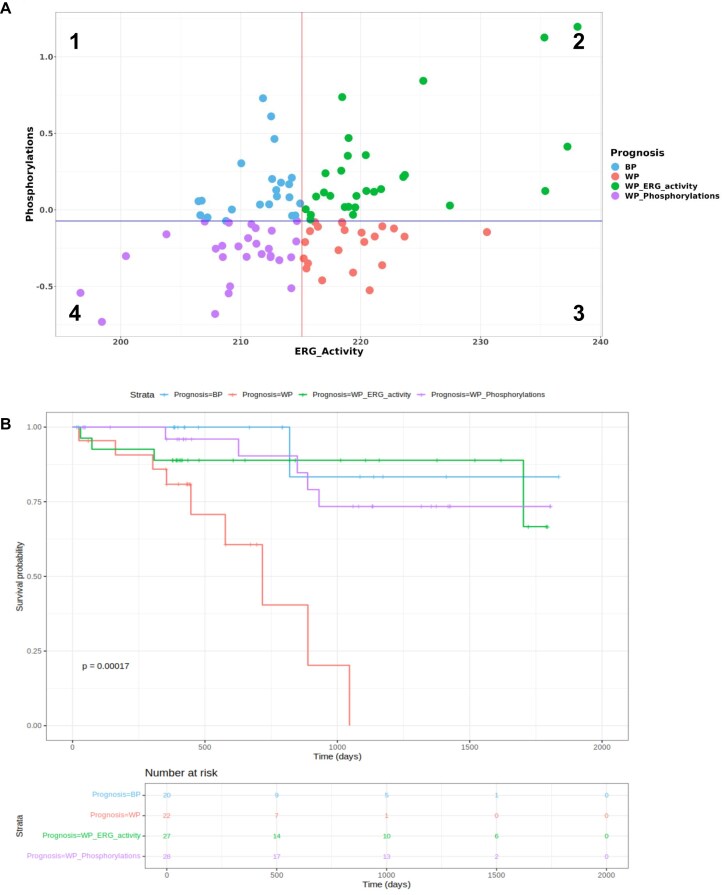
(**A**) Scatterplot of ERG activity scores based on the expression of its targets (*x*-axis) versus the phosphorylation scores that predict its activity (*y*-axis). The solid lines correspond to the median values of the *x*- and *y*-axis. The four quadrants of the plot identify patients for which the activity of ERG is well predicted by the phosphorylation score (2 and 4) versus patients for which this correlation is lost (1 and 3). (**B**) Kaplan–Meier analysis of different groups of patients as defined by the activity of ERG, based on the expression of its targets, and the phosphorylation events that predict its activity.

The phosphorylation predictive of ERG activity involves a set of proteins participating in the VEGFA–VEGFR2 signaling (WP3888) pathway annotated in the WikiPathways database [31], which has a central role in angiogenesis. To integrate the information deriving from the gene expression and phosphoproteomic data we plotted separately the activity of ERG as a function of the phosphorylation levels of the proteins that predict their activity. As expected, for most patients these two quantities are correlated and therefore they capture mostly the same information (Fig. 6A, quadrants 2 and 4 in the plot). However there are two groups of patients (Fig. 6B, quadrants 1 and 3 in the plot) for which the activity of ERG is not associated with the phosphorylation levels of its upstream and downstream proteins. Interestingly in these two groups, the activity of ERG is much more strongly associated with survival, with the patients having lower or higher activity of ERG demonstrating markedly better and worse outcomes respectively. This observation suggests that the integration of these two types of data is able to better capture the underlying biology of ERG and its relationship with outcome.

The results of the Kaplan–Meier survival analysis show that having lower ERG activity scores is associated with a significantly better prognosis. Likewise, having higher values of phosphorylations for the positive coefficient group of ERG, results in better outcomes (see Fig. 6B). The association between ERG and survival when combining transcriptomic and phosphoproteomic data is very strong (*P*-value = 0.00017)

**Figure 7.**
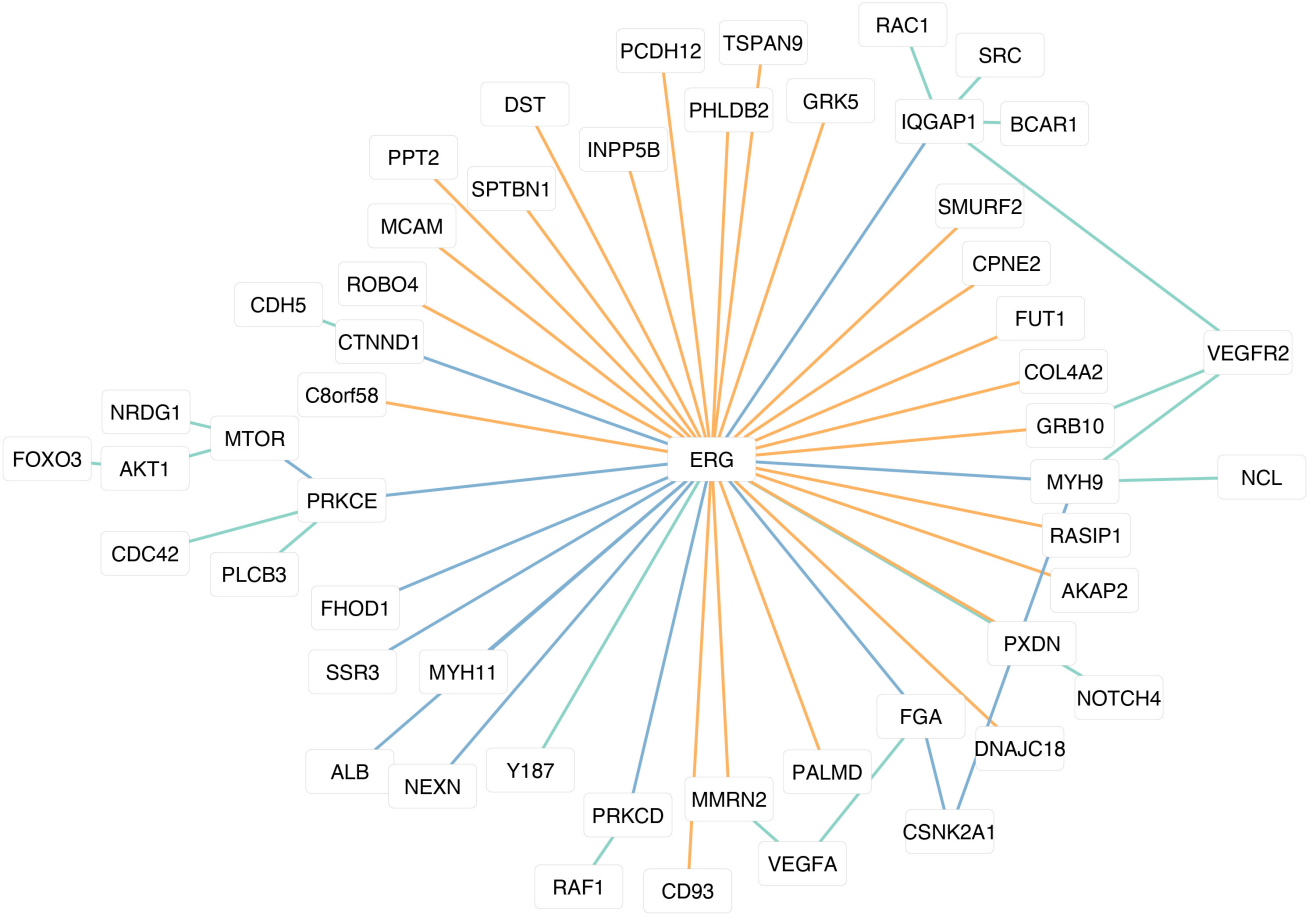
Graph representation of the ERG regulatory network analyzed in this work. The color of the edges indicates the relationship between ERG and the other node. Orange edges represent direct target genes of ERG. Blue edges represent LASSO positive coefficients predictive of ERG activity. Green edges connect members of the VEGFA-VEGFR2 pathway that interact with other nodes of the ERG network.

To develop an integrated model of the activity of ERG and the phosphorylation of upstream and downstream proteins, we built a network comprising the interactions described in the previous paragraphs (see Fig. 7). The network was built with Cytoscape [50], and the edges indicate the relationship between the nodes. The kinases CSNK2A1 and MTOR were added to the network as they are responsible, together with PRKCE, for the phosphorylation of the sites that are predictive of ERG activity. CSNK2A1 phosphorylates MYH9 on Ser1943 [43] and FGA on Ser609 [42], while MTOR phosphorylates PRKCE on Tyr710 [40] as reported in PhosphoSitePlus [38] (see Fig. 7).

**Figure 8.**
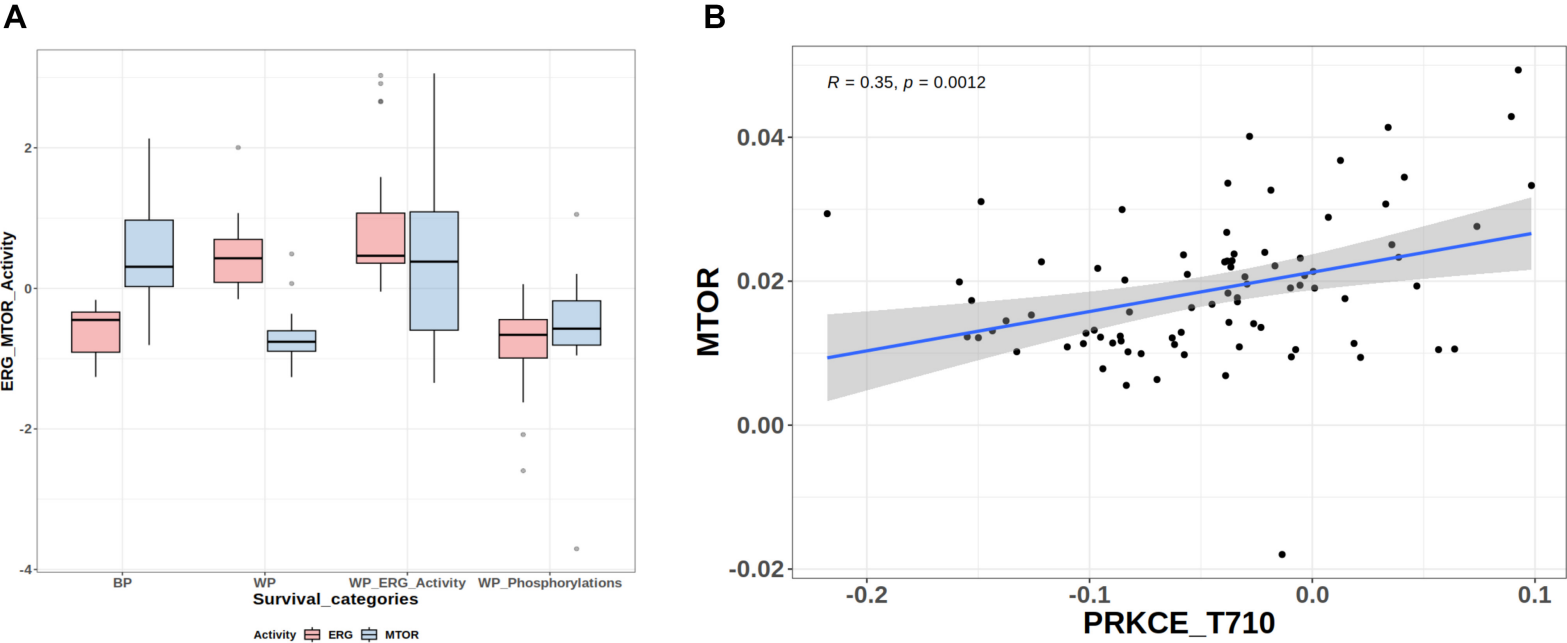
Kinase activity scores for the four patient subsets defined by the activity of ERG (Fig. 6B). The result shows a concordant pattern with the result in Fig. 6B. Correlation between kinase activity scores (*y*-axis) and phosphorylation levels of their target substrates (*x-*axis), expressed as log2 fold change. All phosphorylation levels are positively and significantly (*P*-value ≤ 0.05) correlated with the activity of the corresponding kinase.

Accordingly, we used the RoKai tool to investigate the activity of the PRKCE, CSNK2A1, and MTOR kinases in the four patient groups that were used for the Kaplan–Meier analysis in Fig. 6. Interestingly, the activity of all three kinases is different in the worst versus best prognosis groups, in agreement with the activity of ERG itself. Moreover, the activity of the kinases as defined in this analysis is correlated with the fold change in their target phosphorylation sites (as reported in PhosphoSitePlus) (Fig. 8A and Supplementary Fig. S5). This analysis suggests that this network of kinases and phosphorylation sites is a key regulator of the activity of ERG, which is itself strongly associated with patient survival. Thus, the relationship between phosphorylations and TF activity is maintained when looking at the kinase regulatory layer (see Fig. 8B and Supplementary Fig. S4).

## Discussion

TFs are key regulators of gene expression programs and ultimately govern cell fate. As such, their importance in oncogenesis is well recognized. TFs such as MYC [51], STAT3 [52], and FOXO3 [53] as well as ERG [35] and RUNX1 [54] are established drivers of oncogenic processes. Moreover, mutations in enhancers, which represent the elements through which TFs regulate the expression of target genes, have been documented in multiple cancers, such as in prostate cancer [55], multiple myeloma, and breast cancer [56]. In this study, we set out to develop a novel approach to comprehensively characterize TF activity through the integration of multiple data sources, with the goal of identifying TFs whose activity can be used to stratify patient outcomes in lung cancer. To this end, we performed an extensive re-analysis of a previously published dataset including transcriptomic and phospho-proteomic data for a cohort of 100 NSCLC patients [19]. The main goal of the analysis was to characterize TF activity by integrating multiple lines of evidence, including the activity of their predicted targets, as inferred from gene expression data, the evidence for directed binding of enhancers regulating said targets, and the relationship between the activity of the TF and the phosphorylation of proteins that participate in the same biological pathways, both upstream and downstream. Our analysis identified two TFs (FOXO3 and ERG), whose activity, as inferred from gene expression and genomic data, stratifies patient outcomes, both individually and in combination. When using phosphoproteomic data to assess the relationship between TF activity and the broader signaling state upstream and downstream of the TF, we identified seven TFs whose activity can be predicted from specific phosphorylation events and where the phosphorylation events by themselves can be used to stratify patient outcomes (ELF1, ERG, IRF1, SPI1, ELF2, IRF5, and YBX1). We focused our subsequent analyses on ERG, as this TF was significantly associated with outcome both when its activity was assessed through the lens of transcriptomic as well as phosphoproteomic data. ERG is well known in prostate cancer for its role as a fusion gene product [35], and is also implicated in Ewing sarcoma [34], and in acute myeloid leukemia [57]. This TF is involved in several processes, including angiogenesis and cell proliferation, and also participates in the VEGFA–VEGFR2 pathway [31]. Integration of transcriptomic- and phosphoproteomic-based ERG activity scores resulted in the identification of a patient subset with markedly worse outcomes. Interestingly, the activity of three kinases (PRKCE, CSNK2A1 and MTOR) that are responsible for phosphorylation events that are predictive of ERG activity was also markedly different in the patients with best and worst prognosis.

The main limitation of our study is that the applicability of the workflow described here is restricted to datasets that include both transcriptomic and phosphoproteomic data. Due to the costs and logistical challenges involved, only a few datasets of this type are available. This fact also precludes a complete validation of our results in a completely independent dataset. Our analysis is based on a limited sample size of 100 patients from a single cohort. This raises the possibility that some of our findings may be due to overfitting to the available data and may be biased by the characteristics of the specific cohort utilized. If that were the case, they would not generalize to an independent dataset. These limitations notwithstanding, our analysis demonstrates the usefulness of combining multiple analysis modalities to derive a comprehensive model of TF activity, which enabled us to identify strong relationships with patient outcomes that were not reported in the original analysis of these data.

## Supplementary Material

lqaf068_Supplemental_Files

## Data Availability

For reproducibility, the pipeline we developed to perform LASSO regression and the *corto* MRA analysis is available in the GitHub repository, https://github.com/chiaraCarrino/TF-Activity-Omics-Integration, and in Zenodo, https://doi.org/10.5281/zenodo.15363511. The original transcriptomic and phosphoproteomic datasets analyzed during the current study was generated by the National Cancer Institute Clinical Proteomic Tumor Analysis Consortium (CPTAC) and is available in the Genomic Data Commons repository (https://www.ncbi.nlm.nih.gov/projects/gap/cgi-bin/study.cgi?study_id=phs001287.v5.p4) and in the Proteomic Data Commons (PDC) repository: (https://cptac-data-portal.georgetown.edu/cptac/s/S056). Super Enhancers data on the hg38 human genome reference are available via the SEanalysis website (https://bio.liclab.net/SEanalysis/download). Transcription factor annotations are available in Dorothea (https://saezlab.github.io/dorothea/), The Human Transcription Factors database (http://humantfs.ccbr.utoronto.ca/), and ChEA (https://maayanlab.cloud/Harmonizome/dataset/CHEA±Transcription±Factor±Targets).
