# Supplementary material for: Integrating gene expression, genomic, and phosphoproteomic data to infer transcription factor activity in lung cancer
Source: NAR Genom Bioinform. 2025 May 30;7(2):lqaf068. doi: 10.1093/nargab/lqaf068 (PMC12123410; doi:10.1093/nargab/lqaf068)
Supplement: lqaf068_Supplemental_Files [file lqaf068_supplemental_files.zip › Supplementary Material.pdf]

Supplementary figures

1. Transcription factor activity and metadata analysis in lung cancer patients
2. Kaplan-Meier survival analysis of transcription factor activity
3. Kaplan-Meier survival analysis of ERG and FOXO3 transcription factor activity
4. Analysis of ERG activity and kinase activity in different survival groups
5. Kinase activity compared to substrate's phosphorylation values in patients
6. Validation of TF activity scores in a different cohort

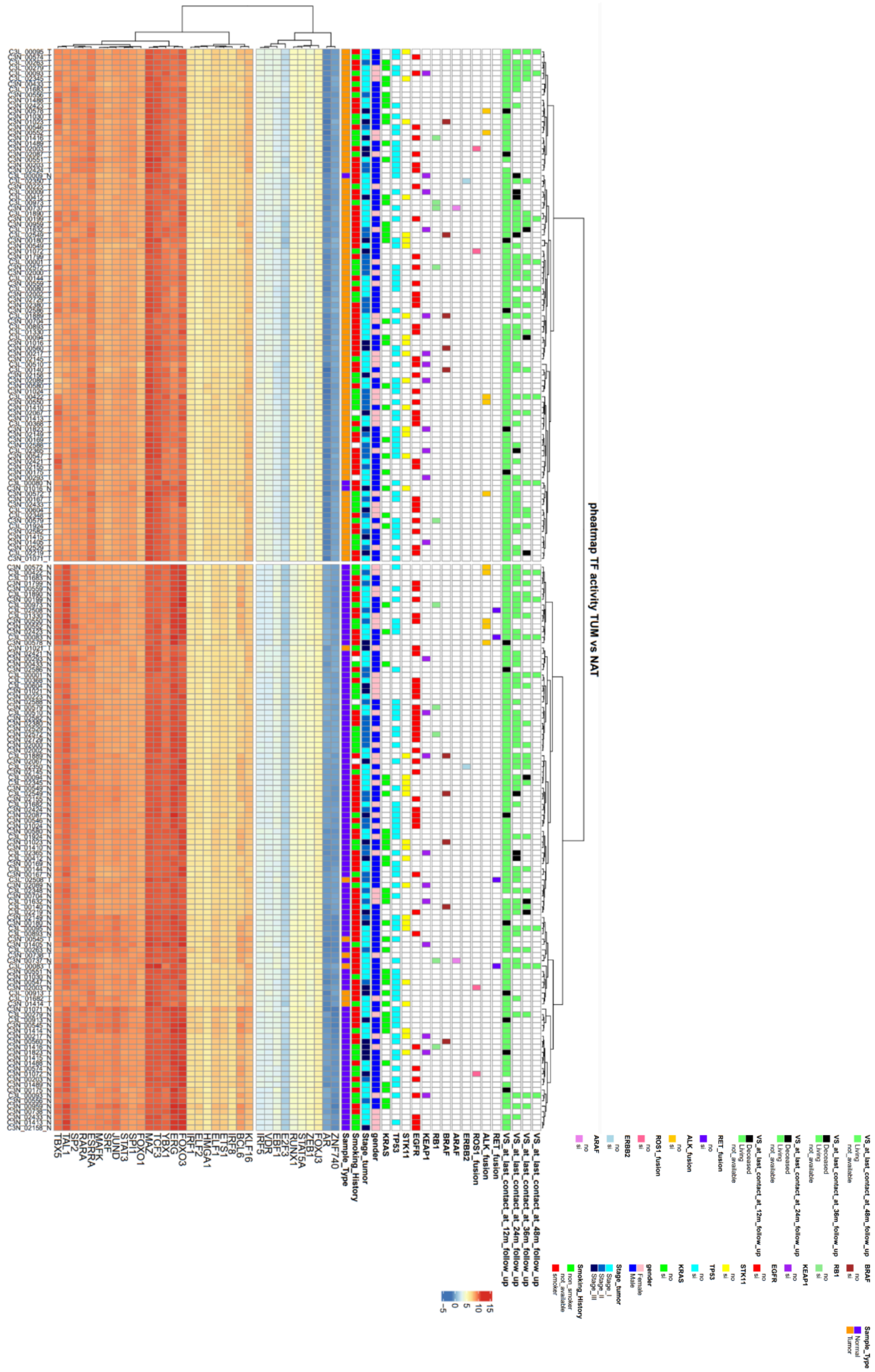

Supplementary Figure 1 | Transcription factor activity and metadata analysis in lung cancer patients Heatmap showing activity values normalized by the number of targets for transcription factors (rows) across all the samples (columns). The top portion of the heatmap indicates the clinical features of the patients. The feature "Sample_type" refers to NAT and tumor samples. Sample metadata employed is available in Supplementary Table 4.

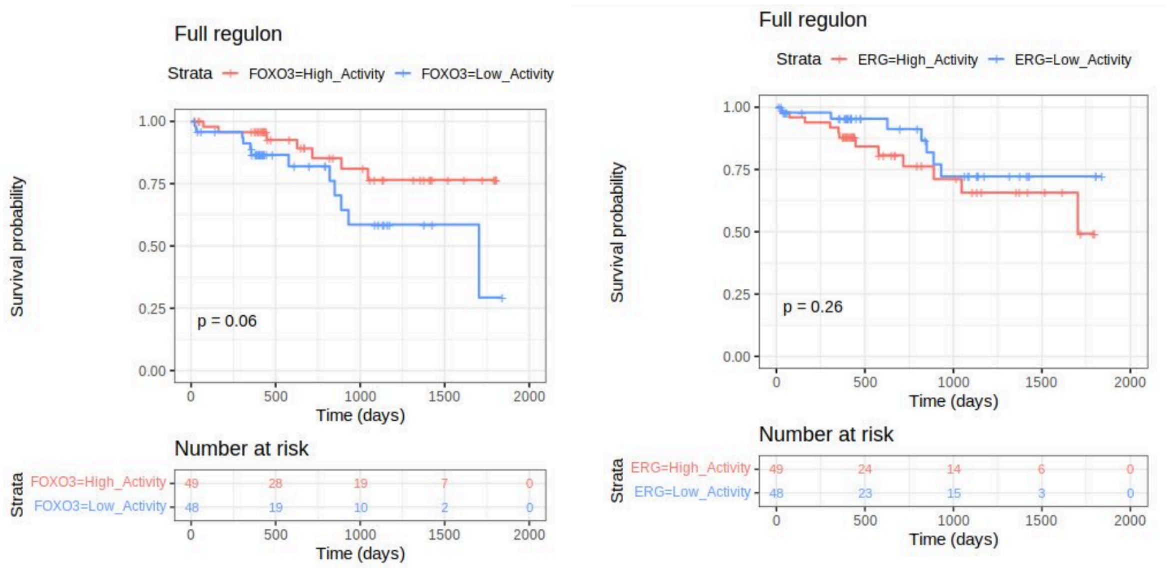

Supplementary Figure 2 | Kaplan-Meier survival analysis of transcription factor activity

Kaplan-Meier survival curves for the two TFs whose activity in tumor samples is significantly associated with patient survival. Patient strata (high vs low) were defined according to the median of the TF activity scores. The top two curves refer to ERG and the bottom two, to FOXO3. The plots correspond to the activity scores calculated from all the target genes, as reported by *corto*.

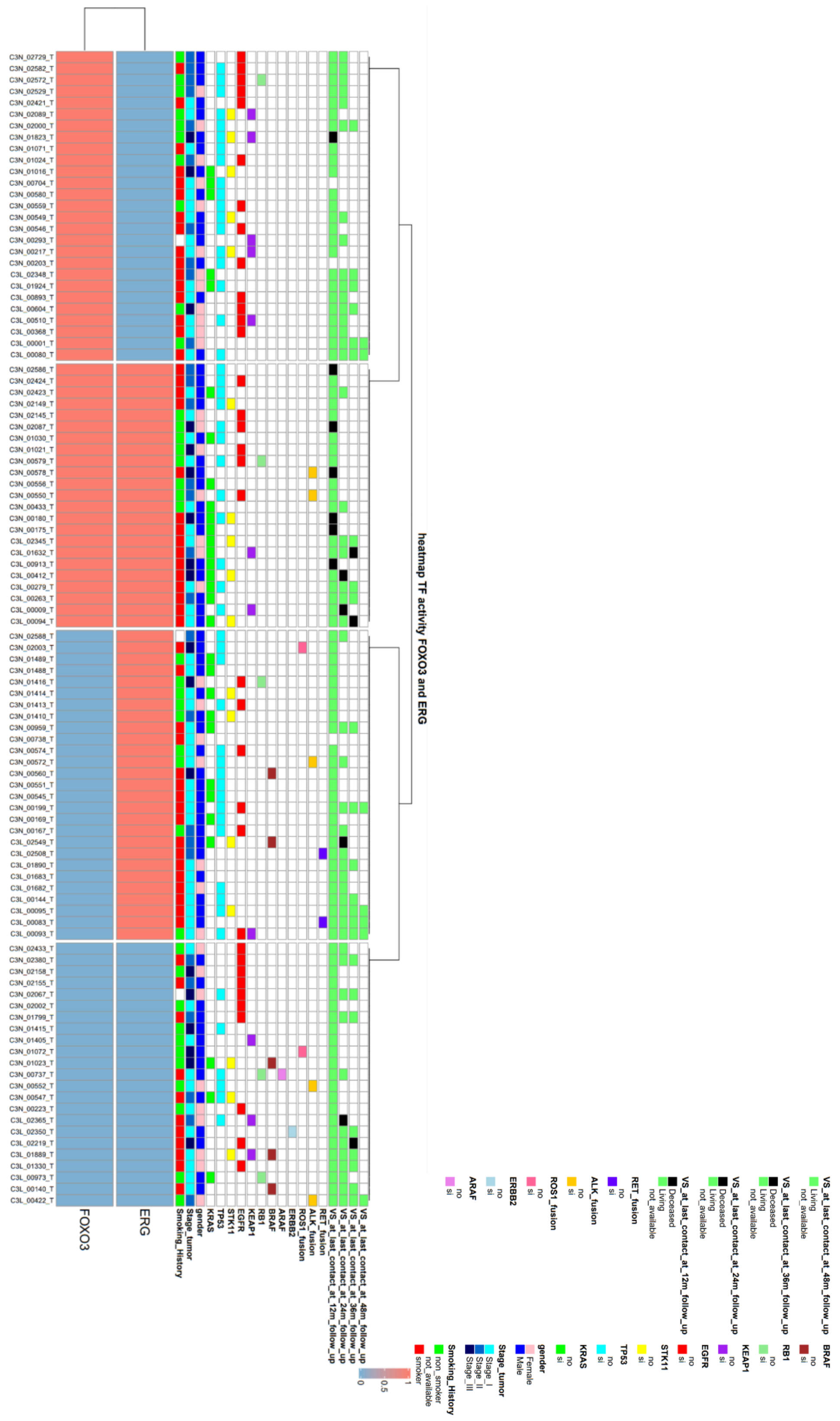

Supplementary Figure 3 | Kaplan-Meier survival analysis of ERG and FOXO3 transcription factor activity

Heatmap displaying the grouping of patients (columns) according to the worst (red) and best (blue) prognosis groups defined by the individual TFs (ERG and FOXO3) indicated in the row.

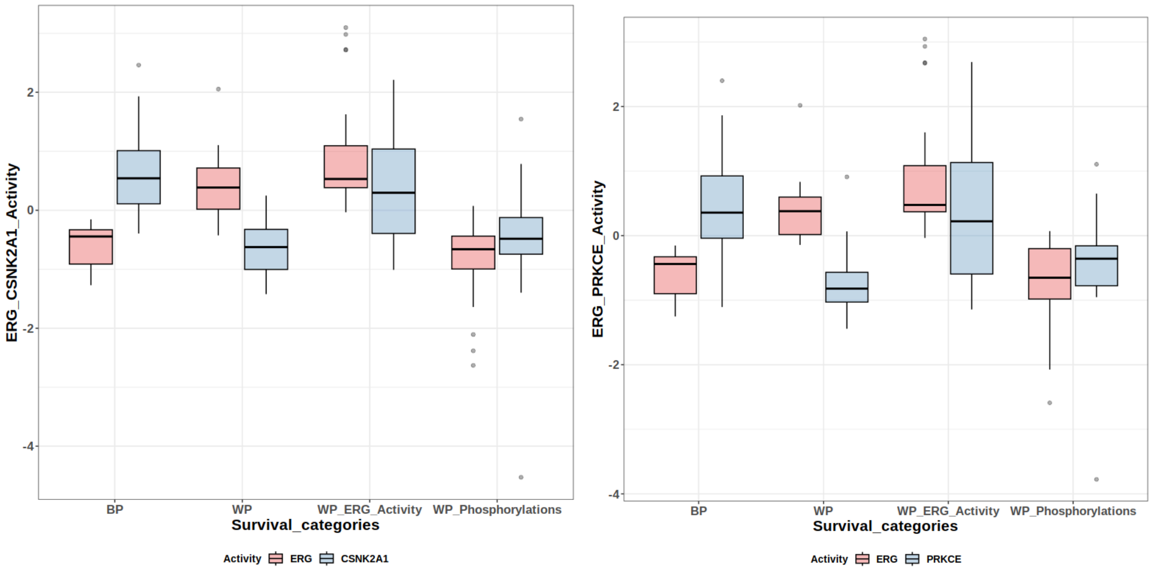

Supplementary Figure 4 | Analysis of ERG activity and kinase activity in different survival groups
Kinase activity scores for the four patient subsets defined by the activity of ERG.

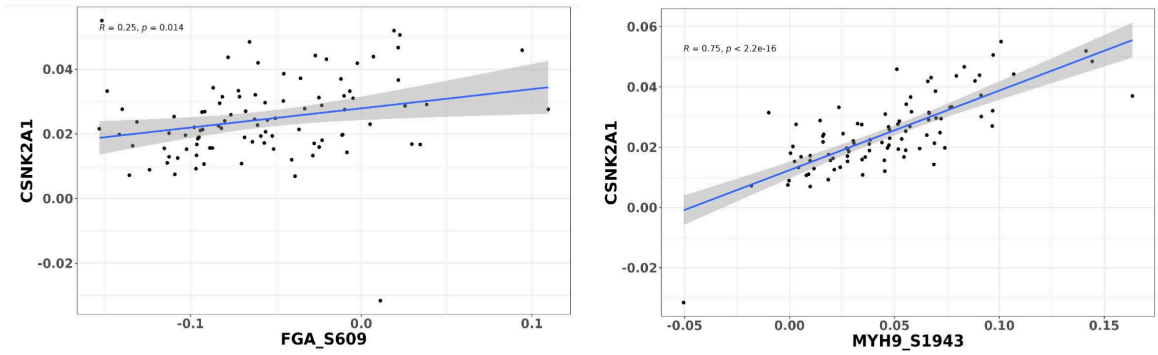

Supplementary Figure 5 | Kinase activity compared to substrate's phosphorylation values in patients
Correlation between kinase activity scores (y axis) and phosphorylation levels of their target substrates (x axis), expressed as log2 fold change. All phosphorylation levels are positively and significantly (p-value<=0.05) correlated with the activity of the corresponding kinase.

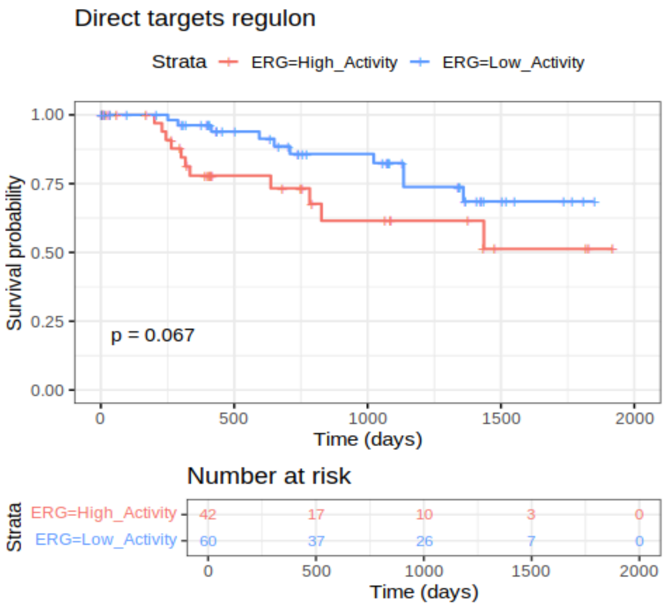

Supplementary Figure 6 | Validation of TF activity scores in a different cohort

We conducted a survival analysis based on a cohort of lung cancer patients from The Cancer Genome Atlas (TCGA) with no prior treatment. The cohort consisted of 102 patients, for whom the activity score was calculated following the methodology outlined in the main manuscript. To calculate a sample-specific transcription factor activity score, we computed, for each sample, the sum of the variance-stabilized expression levels of its direct positive target genes, subtracting the sum of the expression levels of its negative target genes. The results border on significance (p=0.067), which we think is an interesting finding, given the heterogeneity of the datasets and the patient populations, and the fact that we cannot reproduce our entire analytical pipeline as discussed above.
